# New Insights Into Permeation of Large Cations Through ATP-Gated P2X Receptors

**DOI:** 10.3389/fnmol.2018.00265

**Published:** 2018-07-31

**Authors:** Laurie Peverini, Juline Beudez, Kate Dunning, Thierry Chataigneau, Thomas Grutter

**Affiliations:** CNRS, CAMB UMR 7199, Équipe de Chimie et Neurobiologie Moléculaire, Université de Strasbourg, Strasbourg, France

**Keywords:** LGICs, P2X receptors, dilation, spermidine, ion permeation

## Abstract

The permeability of large cations through the P2X pore has remained arguably the most controversial and complicated topic in P2X-related research, with the emergence of conflicting studies on the existence, mechanism and physiological relevance of a so-called “dilated” state. Due to the important role of several “dilating” P2X subtypes in numerous diseases, a clear and detailed understanding of this phenomenon represents a research priority. Recent advances, however, have challenged the existence of a progressive, ATP-induced pore dilation, by demonstrating that this phenomenon is an artifact of the method employed. Here, we discuss briefly the history of this controversial and enigmatic dilated state, from its initial discovery to its recent reconsideration. We will discuss the literature in which mechanistic pathways to a large cation-permeable state are proposed, as well as important advances in the methodology employed to study this elusive state. Considering recent literature, we will also open the discussion as to whether an intrinsically dilating P2X pore exists, as well as the physiological relevance of such a large cation-permeable pore and its potential use as therapeutic pathway.

## Introduction

For most ion channels, ion selectivity remains stable over time once the pore has opened, allowing small metal ions, such as Na^+^, K^+^ and Ca^2+^ to flow across the cell membrane. However, a few channels, namely TRPV1 (Chung et al., 2008), TRPV2 (Nabissi et al., 2013; Zubcevic et al., 2018), TRPA1 (Banke et al., 2010), acid-sensing ion channels (ASICs; Lingueglia et al., 1997; de Weille et al., 1998) and ATP-gated P2X receptors (Khakh and Lester, 1999; Virginio et al., 1999a) exhibit a striking increase in their permeability to larger cations, such as fluorescent dyes or synthetic organic molecules. This phenomenon was initially thought to occur through a time-dependent change of their ion selectivity upon repeated stimulation, a process known as “pore dilation.” However, recent advances have challenged the idea of a slow dynamic change in ion selectivity (Puopolo et al., 2013; Li et al., 2015). In the case of P2X receptors, for which this phenomenon was first described several decades ago, pore dilation has failed to be unanimously accepted due to the increasing emergence of conflicting studies, and alternative mechanisms have been tentatively suggested (Jiang et al., 2005; Rokic and Stojilkovic, 2013; Wei et al., 2016). Given the importance of these ligand-gated ion channels in various physiological and pathological processes, including inflammation and neuropathic pain (Khakh and Alan North, 2006; Abbracchio et al., 2009; Khakh and North, 2012; Lemoine et al., 2012; Bernier et al., 2017), a clear and detailed understanding of this unusual process at the molecular level is of utmost importance.

**Figure 1:**
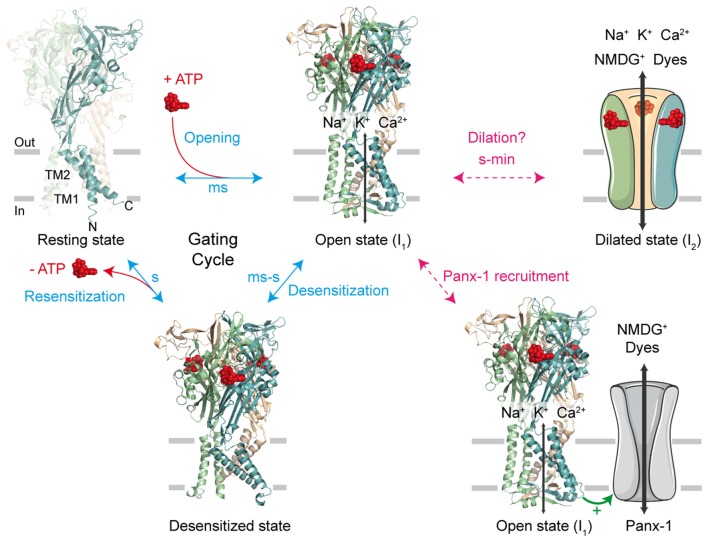
The gating cycle and hypothetical pathways associated to permeation of large cations. In response to ATP binding, the receptor cycles between at least three functional states: the resting state, the open state (sometimes called I_1_ state) and the desensitized state. X-ray structures are from the human P2X3 receptor solved in the apo, resting state (PDB ID 5SVJ), ATP-bound, open channel state (5SVK) and ATP-bound, desensitized state (5SVL; Mansoor et al., 2016). Ribbon structures are viewed laterally from the membrane and each subunit is color coded (one subunit is highlighted in the resting state, in which the N and C termini are indicated). ATP is shown as a space-filling model and the lipid bilayer is symbolized by gray bars. It has been initially suggested that sustained ATP application allows the passage of large cations, such as *N*-methyl-D-glucamine (NMDG^+^) and fluorescent dyes, through two main putative, controversial routes. The first one involves a gradual pore dilation of the open I_1_ state to reach a dilated state (I_2_); the second pathway involves the recruitment of the hemichannel pannexin-1 (Panx-1) by P2X receptor. The two models were suggested not to be mutually exclusive.

First cloned in 1994 (Brake et al., 1994; Valera et al., 1994), the family of P2X receptors is comprised of seven different subunit subtypes (P2X1-P2X7). A functional receptor is composed of three subunits, which are assembled as homo- or heterotrimers (Saul et al., 2013). Each subtype monomer shares a common architecture: two transmembrane domains (named TM1 and TM2) linked by a large, multi-glycosylated and disulfide bridge-containing extracellular domain, and intracellular C- and N-termini (Kawate et al., 2009; Figure 1). There are three ATP-binding sites which are found within the extracellular domain, positioned in cavities at the interface of adjacent subunits (Chataigneau et al., 2013; Habermacher et al., 2016a). In response to ATP binding, P2X receptors cycle between a number of different allosteric conformational states for which X-ray structures are now available (Kawate et al., 2009; Hattori and Gouaux, 2012; Mansoor et al., 2016; Figure 1). Initial ATP binding to the resting, closed channel state triggers a conformational change, resulting in the displacement of all six transmembrane helices and subsequent opening of the transmembrane pore (Li et al., 2008; Kracun et al., 2010). This transition (termed “gating”) usually takes place on the millisecond time scale and allows the small metal cations Na^+^, K^+^ and Ca^2+^ to pass through the open pore (sometimes called I_1_ state) according to their electrochemical gradient (North, 2002). Sustained application of ATP then leads to the inactivation of the pore (with the exception of P2X7), a process called desensitization, in which ion flux is terminated despite the fact that ATP remains bound to the receptor. ATP dissociation from these states reverts the pore to the initial closed state, from which the receptor is able to undergo further gating cycles upon re-activation (Figure 1).

To account for the gradual increase of permeability to larger cations, several mechanistic pathways linked to the P2X gating cycle have been proposed (Jiang et al., 2005; Rokic and Stojilkovic, 2013; Wei et al., 2016). For one of these routes, a putative dilated state (called I_2_ state) corresponding to a progressive pore expansion of the open I_1_ state following sustained ATP application has been suggested (Khakh and Lester, 1999; Virginio et al., 1999a; Figure 1). However, recent studies have seriously challenged this pore dilation hypothesis, by demonstrating that this phenomenon is rather an artifact of the method used to measure ion permeability change. In this review, we will discuss briefly the historical emergence of the controversial dilated state in the P2X family, as well as the recent important advances in the methodology employed that have led to the reconsideration of pore dilation. Finally, we will open the discussion to new physiological and therapeutic perspectives.

## Brief History of the Increase of Permeability

The ability of ATP to permeabilize membranes from different cells, such as mast cells, macrophages and transformed fibroblasts, was first discovered in the late 70s by demonstrating that the membrane permeability of cells gradually increases with increasing concentrations of ATP (Cockcroft and Gomperts, 1979). Besides small inorganic cations, larger organic molecules, such as carboxyfluorescein (376 Da), ethidium bromide (394 Da), lucifer yellow (444 Da) and fura-2 (636 Da) were also shown to be able to cross membranes following ATP application. However, solutes of higher molecular weight, such as Trypan Blue (872 Da), Evans Blue (961 Da) and inulin (more than 5000 Da) did not permeate the membrane (Steinberg et al., 1987; Steinberg and Silverstein, 1989), suggesting that molecules exceeding a certain radius cannot pass. At this point in time, the protein responsible for this behavior was unknown, and additionally, the observed “pore formation” features largely varied according to the cellular subtypes studied (Heppel et al., 1985).

Following these observations, and the discovery of ATP-activated purinergic receptors (P2X and P2Y; Burnstock, 1990), the passage of such large molecules across cell membranes was attributed to the fact that those cell types, in particular immune cells, express an unusual P2 receptor with an apparently non-selective pore. This was initially classified as the P2Z receptor: the “pore-forming” protein was thus named (Gordon, 1986; Abbracchio and Burnstock, 1994). P2Z receptors were then extensively studied for their ability to allow the permeation of large organic molecules into macrophages through a “macropore” (Nuttle and Dubyak, 1994), leading to cytolysis (Di Virgilio, 1995). The link with a putative P2X-related mechanism arose from the identification of the close proximity between P2Z receptor and P2X family receptor sequences. This similarity led to the re-classification of P2Z as the P2X7 receptor subtype (Surprenant et al., 1996). A few years later, two seminal articles reported that other P2X subtypes, the P2X2 and P2X4, also exhibit a striking increase in the permeability of large cations following several seconds of ATP application (Khakh et al., 1999; Virginio et al., 1999b). The concept of P2X “pore dilation” was born and multiple research groups have since confirmed and extended the concept to homomeric and heteromeric receptors (e.g., P2X2/3 and P2X2/5) expressed either in recombinant systems or in native tissues (Khakh et al., 1999; Virginio et al., 1999b; Yan et al., 2008; Compan et al., 2012; Browne et al., 2013). However, certain P2X subtypes, namely P2X1 and P2X3 which are fast desensitizing receptors, were classified as “non-dilating” channels as there was no evidence for an apparent increase in the permeability to large cations. Moreover, distinctive behaviors have been observed for P2X7 receptor splice variants, of which there are nine in humans, classified from P2X7B to P2X7J, and four in rodents (P2X7B, P2X7C, P2X7D and P2X7K); P2X7A is the full-length common P2X7 subunit (Rassendren et al., 1997; Cheewatrakoolpong et al., 2005; Sluyter, 2017). Some of these variants show an altered permeability for large cations. For instance, P2X7B, a variant bearing a largely truncated C terminus, is considered “non-dilating” (Cheewatrakoolpong et al., 2005; Adinolfi et al., 2010). On the contrary, P2X7K, which features a modified TM1 sequence as well as a more lipophilic N terminus, has been shown to be constitutively and immediately dilated (Nicke et al., 2009; Xu et al., 2012).

## Methods and Interpretations Leading to the Controverted Dilated State

Two main experimental approaches have been used to monitor the apparent gradual increase in large cation permeability. In the first approach, the cellular uptake of fluorescent dyes, such as YO-PRO-1 or ethidium bromide, is observed as a function of time. Dye uptake usually develops several seconds after ATP application. As these cationic dyes become fluorescent when bound to DNA, this method is a simple and direct read-out of the passage of large molecules following P2X activation. Studies involving cysteine reactive compounds, such as methanethiosulfonate (MTS) reagents, have also shown the ability of larger sized molecules to pass into the cell (Browne et al., 2013). This method has been employed for P2X2, P2X4 and P2X7 receptors (Khakh et al., 1999; Virginio et al., 1999a,b; Yan et al., 2008).

In the second approach, the permeability of large cations is measured by patch-clamp electrophysiology (Khakh et al., 1999; Virginio et al., 1999b). In this indirect method, reversal potentials (E_rev_) are measured in bi-ionic solutions, where *N*-methyl-D-glucamine (NMDG^+^), a synthetic large organic cation, is the sole permeating ion present outside the cell (NMDG^+^_out_) and Na^+^ is the sole permeating cation present inside the cell (Na^+^_in_). According to the Goldman-Hodgkin-Katz voltage equation, a shift in E_rev_ signals a change in membrane permeability to one or both cations provided the concentrations of ions on either side of the membrane remain unchanged. The gradual and positive shifts in E_rev_ measured during long applications of ATP have been interpreted as a time-dependent change in the relative permeability of NMDG^+^ relative to ${\text{Na}}^{+}({\text{P}}_{{\text{NMDG}}^{+}}+{\text{P}}_{{\text{Na}}^{+}})$. At the beginning of extracellular ATP application, the agonist opens a pore that is initially more permeable to Na^+^ than NMDG^+^, but during sustained activation of the channel, E_rev_ shifts towards less negative values that signify a dramatic increase in the permeability to NMDG^+^ (Khakh et al., 1999; Virginio et al., 1999b). This method has been extensively employed to probe shifts in E_rev_ for many P2X receptors, such as P2X2, P2X2/3, P2X2/5, P2X4 and P2X7 (Khakh et al., 1999; Virginio et al., 1999a,b; Compan et al., 2012).

The fact that shifts of E_rev_ and dye uptake occur on the same time scale has led to the belief that a common mechanism would be at work. Two main hypotheses have then emerged (Figure 1). One hypothesis involves the recruitment of an auxiliary protein by P2X receptors. This protein is pannexin-1 (Panx-1), a hemichannel that is responsible for the passage of larger molecules. This hypothesis is supported by the observation that P2X permeability to large cations is inhibited when inhibitors of Panx-1 are applied (Pelegrin and Surprenant, 2006). In addition, colchicine, a drug that disrupts the cytoskeleton, has been shown to affect YO-PRO-1 uptake into cells transfected with P2X2 or P2X7 subtypes, but not the permeability for small cations. This would indicate that these two types of permeability result from different pathways (Marques-da-Silva et al., 2011).

On the other hand, a second hypothesis postulates the existence of an intrinsic “pore dilation” mechanism, whereby the P2X pore itself undergoes a slow conformational change. This would lead to a physical expansion in the diameter of the P2X ion pore from the I_1_ state to the putative I_2_ dilated state, allowing the passage of larger molecules. This “pore dilation” mechanism has been extensively studied by site-directed mutagenesis, patch-clamp electrophysiology, fluorescent dye uptake and use of chemical reagents (Khakh et al., 1999; Virginio et al., 1999a,b; Eickhorst et al., 2002; Khakh and Egan, 2005; Browne et al., 2013). Moreover, it has been shown that cells which do not express Panx-1 nonetheless exhibit “dilating” properties and, in agreement with this hypothesis, overexpression or inhibition of Panx-1 by carbenoxolone does not affect dilation of P2X2 receptors expressed in HEK-293 cells (Chaumont and Khakh, 2008; Yan et al., 2008).

As evidence has been found both in support of and against each hypothetical pathway, the question has been raised as to whether these two distinct mechanisms may in fact co-exist (Jiang et al., 2005; Cankurtaran-Sayar et al., 2009). One mechanism would be dedicated to NMDG^+^ permeability and the other to larger molecules such as YO-PRO-1 and even occasionally the permeation of organic anions (Browne et al., 2013). Moreover, interactions with other proteins may play a role. For instance, cytoskeletal proteins have been described as important for pore dilation in P2X7 receptor subtype (Kim et al., 2001; Gu et al., 2009). In addition, characterization of the apparent “pore dilation” has been shown to be dependent on cell types used for experiments as well as expression levels of receptors (Fujiwara and Kubo, 2004). Of greatest importance is the fact that dynamic changes of unitary conductance following prolonged ATP application have never been observed at the single channel level (Ding and Sachs, 1999; Riedel et al., 2007b), thus bringing into question the reality of an intrinsic “pore dilation”.

## Changing the Paradigm

In 2015, the team of K. Swartz offered an alternative explanation of the change in E_rev_ measured in bi-ionic ${\text{NMDG}}_{\text{out}}^{+}/{\text{Na}}_{\text{in}}^{+}$ solutions (Li et al., 2015). The authors elegantly demonstrate in the P2X2 receptor that shifts in E_rev_, although very real, are not caused by a time-dependent change in channel permeability, but rather by a dramatic and unappreciated change of the intracellular ion concentrations, which may vary by more than 100 mM throughout the course of the experiment. Combining electrophysiology and mathematical modeling, the authors were able to convincingly demonstrate that prolonged ATP activation leads to a depletion of intracellular Na^+^, from 140 mM to around 20 mM, and accumulation of NMDG^+^, from 0 mM to over 200 mM. They further showed that physical parameters such as channel densities, pore conductance, cell volume, and access resistance (*R*_access_) between the cell and the pipette electrode, may directly change ion concentrations inside the cell following sustained exposure to ATP. Because of these changes, the Goldman-Hodgkin-Katz voltage equation cannot be used to reliably determine the relative permeability of ions. This study therefore proved that the time-dependent shift in the E_rev_ measured in bi-ionic solutions is an artifact of the method employed, and that extreme caution must be taken when measuring permeability changes during whole-cell patch-clamp recordings.

This outstanding article seriously challenges the pore dilation paradigm which held true for almost two decades. However, it is important to emphasize that the study did not question the ability of NMDG^+^ to permeate through the activated P2X pore, as initially suggested by the seminal articles (Khakh et al., 1999; Virginio et al., 1999b), but that this NMDG^+^ permeation occurs soon after ATP activation. As a direct proof of this assumption, Li et al. (2015) showed that whole-cell currents carried by NMDG^+^ using symmetrical solutions (whereby both intracellular and extracellular solutions contain NMDG^+^) developed immediately following ATP application. These observations indicate that a gradual pore dilation mechanism is not necessary to account for NMDG^+^ permeation.

## A New Mechanistic Point of View

The publication of Li et al. (2015) opens an appealing hypothesis: the dimensions of the pore itself may be directly adequate for the direct passage of NMDG^+^ following several milliseconds of ATP application. From a mechanistic point of view, the I_1_ state would represent the most parsimonious hypothesis, provided the diameter of the narrowest part of its open pore is sufficiently wide to allow the passage of such large molecules. We have investigated this possibility by combining single-channel recordings, molecular modeling and the use of photo-switchable tweezers (Harkat et al., 2017), a recently developed technology that allows identification of the molecular motions involved in channel activation (Habermacher et al., 2016b). By covalently tethering synthetic azobenzene cross-linkers to a pair of selected engineered cysteine residues, these “molecular tweezers” are capable of pulling or pushing on gating elements once azobenzene isomerization is triggered by light irradiation. To provide a read-out of the large cation permeation, we used symmetric NMDG^+^ solutions, where NMDG^+^ was the sole permeating ion. A rapid solution exchange of the extracellular solutions from an NMDG^+^-containing to a Na^+^-containing solution allowed direct comparison of molecular motions involved in pore opening and permeation of NMDG^+^ and Na^+^. We screened several positions near a flexible region of TM2 in the P2X2 receptor, as well as a region of the pore surrounding a kink that was previously identified in channel gating (Habermacher et al., 2016b; Mansoor et al., 2016). Firstly, we identified two types of molecular motions involved in the permeation of large cations: a horizontal expansion of the upper end of TM2 helices and a vertical flexing of the extremities of two adjacent TM2 helices. Surprisingly, we observed no NMDG^+^ permeation for mutants bearing no permeability to Na^+^, suggesting that none of the molecular motions that we monitored can produce a pathway dedicated exclusively to NMDG^+^ permeation. Secondly, for the motions that allowed the passage of both NMDG^+^ and Na^+^, the ratio of the current ${\text{I}}_{{\text{NMDG}}^{+}}{\text{/I}}_{{\text{Na}}^{\text{+}}}$ was around 10%, suggesting that NMDG^+^ follows the same pathway as Na^+^ across the P2X pore but is not able to permeate as easily.

**Figure 2:**
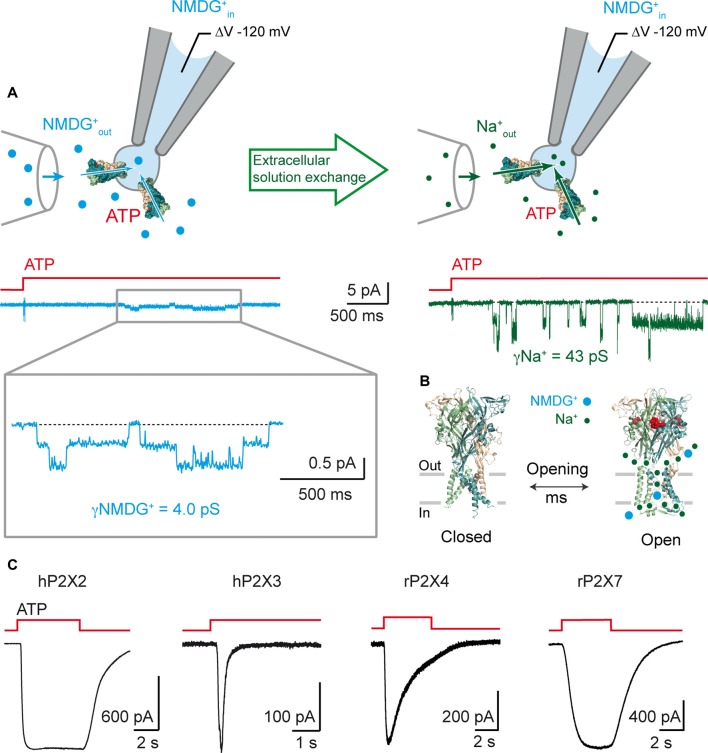
P2X receptors are rapidly permeable to large cations. **(A)** Rapid onset of single-channel NMDG^+^ currents following ATP application. Top: schematic drawing of the outside-out configuration and perfusion glass tube. Note that patch pipette contains NMDG^+^. Bottom: Single-channel currents elicited by 1 μM ATP from an outside-out patch expressing the rat P2X2 receptor. Currents were first recorded in extracellular NMDG^+^_out_ solution (symmetric NMDG^+^_out_/NMDG^+^_in_, left, blue), and then in extracellular Na^+^_out_ solution (Na^+^_out_/NMDG^+^_in_, right, green), following rapid solution exchange. Currents were recorded at −120 mV and filtered at 1 kHz. Inset shows magnification of NMDG^+^ currents filtered at 100 Hz. ATP applications are indicated by red stepped bars. Adapted from Harkat et al. (2017). **(B)** Proposed model in which NMDG^+^ and Na^+^ ions transit through the same open pore at different flow rates following rapid ATP activation (~2.5 *×* 10^6^ NMDG^+^ ions/s at −120 mV with 132.6 mM NMDG^+^, 32 × 10^6^ Na^+^ ions/s at −120 mV with 132.6 mM Na^+^; data taken from Harkat et al. (2017)). **(C)** Whole-cell ATP-gated currents recorded in symmetric spermidine solution at −60 mV from HEK-293 cells expressing the indicated P2X receptors. Traces for hP2X2 and hP2X3 are adapted from Harkat et al. (2017). ATP concentration was 30 μM for hP2X2 and hP2X3, 100 μM for rP2X4, and 1000 μM for rP2X7.

To further support this observation, we recorded ATP-induced single-channel currents from outside-out patches using symmetrical NMDG^+^ solutions (Figure 2A). We not only showed that P2X2 receptors are NMDG^+^-permeable channels, activated on the millisecond timescale, with kinetics similar to those observed when Na^+^ is the primary permeating ion, but we also established the first measurements of the unitary conductance of NMDG^+^ currents (around four pS for several ATP concentrations). This is approximately 10 times lower than that of Na^+^ currents. We further confirmed these experimental data by molecular dynamic simulations carried out on a model of ATP-bound P2X receptors. These computations represent a step forwards in the understanding of the molecular mechanism of NMDG^+^ permeation, which is the result of a conformational and orientational selection process (Harkat et al., 2017). We further validated our methodology by measuring YO-PRO-1 uptake in cells expressing engineered receptors, to discount the presence of possible artifacts caused by the use of non-physiological media for electrophysiological recordings. For the mutants exhibiting light-induced motions leading to a “desensitizing-like” phenotype, we were not able to show fluorescence uptake, but for those associated with a stable opening induced by light, YO-PRO-1 was successfully incorporated into cells, suggesting that desensitization may hinder dye accumulation into cells. However, no YO-PRO-1 uptake was observed during molecular dynamic simulations, which brings back the possibility of two distinct mechanisms for YO-PRO-1 and NMDG^+^ uptake. Alternatively, this could be the result of a limitation of the method used, as it is possible that the sampling time for computations may not have been sufficient to observe the passage of low-conductance YO-PRO-1. Altogether, our data support that the open I_1_ state likely represents the main route for both small and large cations (Figure 2B).

In agreement with data obtained from P2X2 receptors, two recent reports carried out on P2X7 receptors further support an instantaneous permeation of large cations upon ATP activation (Karasawa et al., 2017; Pippel et al., 2017). In the first study, a rigorous and thorough analysis of single-channel currents demonstrate that the unitary conductance of the human P2X7 channel remains stable over time following prolonged ATP application (up to 30 min; Riedel et al., 2007a,b; Pippel et al., 2017). Interestingly, the authors even showed that chemical modification of engineered cysteine side chains with charged MTS reagents increased dramatically the open probability of the channel, with no sign of apparent pore dilation (Pippel et al., 2017). In the second study, the biophysical properties of the “macropore” feature of P2X7 channel have been scrutinized in a synthetic system, in which the functionality of the P2X7 protein has been monitored in the absence of other protein cellular components (Karasawa et al., 2017). To do so, a purified, yet truncated, version of the giant panda P2X7 receptor (pdP2X7-ΔNC) was reconstituted into manufactured liposomes. To distinguish channel activity (I_1_ state) from “macropore” formation (I_2_ state), proteoliposomes encapsulated either Fluo-4, a Ca^2+^-sensing fluorescent probe, or DNA, to which binding of YO-PRO-1 enhances dye fluorescence emission. The authors elegantly and convincingly showed that pdP2X7-ΔNC was not only permeable to YO-PRO-1, but also to Ca^2+^ with the same apparent kinetics, suggesting a common pathway for both small and large cations (Karasawa et al., 2017). Therefore, in a reconstituted system, the purified P2X7 receptor is able to form an intrinsic and immediate dye-permeable pore with no apparent time-lag, a finding that is apparently not compatible with the slow and progressive process of pore dilation.

## Is the Concept of Pore Dilation Dead?

**Figure 3:**
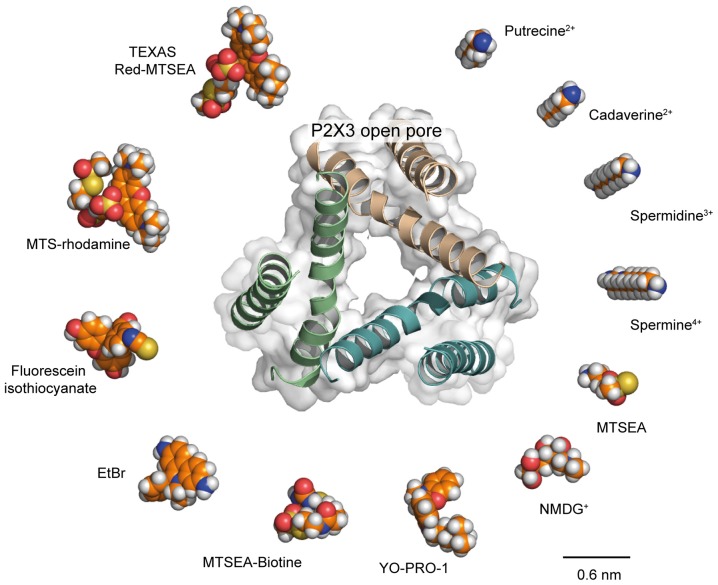
Is the P2X open pore sufficiently wide to allow the passage of large molecules? Comparison of structures of selected organic molecules, shown as space-filling models, scaled to the X-ray structure of the P2X3 open pore (PDB ID: 5SVK), viewed along the three-fold axis of symmetry. Receptor ribbon structure is superimposed to the protein surface. Shown is a clockwise ranking of molecules, from the smallest (putrescine, top right) to the largest dimensions (Texas Red-MTSEA, top left). Valency of cations is also indicated. Recent evidence supporting permeation of NMDG^+^ and spermidine through the P2X2 open I_1_ state (Harkat et al., 2017) suggests that molecules of similar size to these cations should also permeate the P2X open pore. For slightly larger molecules, such as YO-PRO-1, for which their dimensions reach those of the open pore, we propose that such molecules follow the same pathway as Na^+^ across the P2X pore but are not able to permeate as easily. For the largest molecules, such as Texas Red-MTSEA, there is evidence that they cannot enter the P2X2 pore (Li et al., 2010). However, methanethiosulfonate (MTS)-rhodamine, which has a size that is similar to that of Texas Red-MTSEA, has been shown to directly permeate the P2X7 pore (Browne et al., 2013), suggesting the P2X7 open pore is larger than that of P2X2. From a structural point of view, the open state of the P2X3 receptor seems too narrow to allow the passage of MTS-rhodamine. Therefore, either the structure of P2X7 open state is different from that of the P2X3 receptor or other routes specifically dedicated to the permeation of such very large molecules in P2X7 receptors exist. Carbon atoms are indicated in orange; nitrogen atoms in blue, oxygen atoms in red; phosphorus atoms in yellow, and hydrogen atoms in white.

As stated above, recent data now support the hypothesis that the open I_1_ state would be sufficiently wide to allow the passage of relatively large molecules, without the need for an ATP-induced pore dilation. However, comparison of the structure of the largest permeating molecules with recent X-ray structures of the ATP-bound, open state casts doubt on the fact that the open I_1_ state truly represents the sole ion pathway (Figure 3). This is particularly true for P2X7 receptors, for which MTS-rhodamine, a nanometer-sized dye, was shown to penetrate the open channel and block at a cysteine-substituted residue located deeply in the pore (Browne et al., 2013), whereas the structure of the open pore derived from the human P2X3 receptor (the structure of P2X7 open pore is not yet determined) appears to be too narrow to accommodate such a molecule. Interestingly, molecules of similar size, such as Texas Red-MTSEA, cannot enter the P2X2 pore (Li et al., 2015) suggesting that the P2X7 receptor, when activated by ATP, can reach a diameter that is larger than that of the P2X2 receptor (Figure 3). Other routes specifically dedicated to the permeation of such very large molecules may also exist. As stated above, Panx-1 is a candidate, as close proximity between Panx-1 and P2X7 channels has been established in human and mouse macrophages cells (Pelegrin and Surprenant, 2006). Alternatively, there is still a possibility that P2X7 receptors do indeed dilate under certain conditions that remain to be determined.

A finding which may reconcile P2X7 (and perhaps other P2X subtypes) with the concept of “pore dilation” comes from recent studies that emphasize the importance of the lipid composition of the membrane for the passage of large cations through the P2X pore. Although lipids, such as phosphoinositides (Bernier et al., 2008a,b) and cholesterol (Murrell-Lagnado, 2017), seem to be important for the regulation of allosteric states of P2X receptors, this factor has been underestimated in “dilation” studies thus far. However, Kawate and co-workers (Karasawa et al., 2017) have very recently demonstrated in reconstituted liposomes that sphingomyelin and phosphatidylglycerol-containing membranes facilitate pdP2X7-ΔNC-dependent dye uptake, whereas the presence of cholesterol rather inhibits this process. It is thus conceivable that the lipid composition may have a direct impact on the physical diameter of the P2X7 open channel state that would allow the passage of very large molecules (up to 900 Da). Alternatively, a conformational plasticity in the selectivity filter of the P2X pore may allow the passage of large molecules, as very recently shown on TRPV2 ion channel (Zubcevic et al., 2018). Although appealing, these hypotheses need convincing evidence; in particular the structure of the P2X7 open channel state (and also other P2X subtypes) solved in a lipid bilayer will certainly advance this issue.

## Physiopathological Role of Large Cation Permeation

Now that there is no doubt that P2X receptors are permeable to large cations, is there any link between this feature and pathological states? Evidence exists indicating a possible link at least involving the P2X7 receptor, which is arguably of most therapeutic interest due to its implication in a large variety of diseases. Expressed in a range of immune and microglial cells, P2X7 receptors are widely involved in the immune and inflammatory response, notably in the nervous system. Several recent reviews extensively cover the involvement of P2X7 receptors in pathological immune-related conditions, such as neuropathic pain, Alzheimer’s disease, Huntington’s disease and multiple sclerosis to name but a few (De Marchi et al., 2016; Di Virgilio et al., 2017; Savio et al., 2018). A first possible link may involve the ability of Panx-1 channels to allow entry of large cations into the cell, as it has been demonstrated that an increase in extracellular ATP induces the clustering of Panx-1 with P2X7 receptors, leading to their internalization (Boyce et al., 2015). An impairment of this internalization mechanism could contribute to the over-activity of P2X-mediated signaling that may lead to pathophysiological states, such as neuropathic pain (Swayne and Boyce, 2017). Another possible link may involve the P2X7 protein itself. An association between nerve-induced pain behavior (mechanical allodynia) and the P451L mutation found in the mouse *P2rx7* gene has been identified. This mutation impaired “pore formation” by affecting the entry of YO-PRO-1 and ethidium bromide in HEK-293 cells and calcein dye into macrophages, but interestingly the mutation did not affect Ca^2+^ entry (Adriouch et al., 2002; Young et al., 2006; Sorge et al., 2012). Mice in which P2X7 receptors have impaired pore formation as a result of this mutation showed less allodynia than mice with the pore-forming receptors. Moreover, in two independent human chronic pain cohorts, it has been shown that human mutations affecting the amino acid sequence of P2X7 receptor modify the chronic pain sensitivity (Sorge et al., 2012). For instance, in the cohort experiencing post-mastectomy chronic pain, the H155Y single nucleotide polymorphism, which leads to hyperfunctional P2X7 receptors, was associated with an increased pain sensitivity. In contrast, mastectomized women with the hypofunctional mutation R270H of P2X7 receptor, experienced reduced chronic pain (Sorge et al., 2012). These correlations thus clearly suggest that the level of pore activity is related to pathological states and indicate that P2X7 receptors are vital targets for the treatment of diseases such as neuropathic pain. Differences in Na^+^, K^+^ and Ca^2+^ ion flux may trigger such pathological responses, but permeation of large, pathologically relevant cations may also be involved. These large molecules remain to be identified.

## New Directions Concerning the P2X Permeation of Large Cations: The Case of Polyamines

What is the physiological implication of P2X permeability to large cations? To answer to this question, it is first important to consider whether this unusual large cation permeation feature is shared by all members of the P2X family. Before 2017, a striking observation was that fast-desensitizing receptors, namely P2X1 and P2X3 receptors, were apparently unable to allow the entry of larger cations through the pore. By using symmetric solutions, we have provided evidence that P2X3 receptors are actually permeable to NMDG^+^, suggesting that these receptors should be reconsidered as channels permeable to large cations (Harkat et al., 2017). However, NMDG^+^ is a synthetic molecule, and we sought rather to establish the permeability of large, naturally occurring cations through the P2X pore. We have very recently found that spermidine permeates through the human P2X2 and P2X3 receptor ion channels (Harkat et al., 2017). Now we have extended this permeation to the rat P2X4 and P2X7 receptors, suggesting permeation of spermidine may be a common feature shared by many, if not all members of the P2X family (Figure 2C).

Spermidine is a natural, ubiquitously distributed polyamine that is vital for the cell (Guerra et al., 2016; Madeo et al., 2018). It is formed intracellularly and is involved in several important cellular functions, such as growth, division and proliferation. Spermidine is also recognized as an allosteric modulator of many ion channels, including ionotropic glutamate receptors, nicotinic acetylcholine receptor, potassium channels, ASICs and TRPV1 channels (Table 1). For some of these channels, spermidine must exit the cell through dedicated pathways to reach the targeted extracellular sites. One of these such pathways may be the vesicular polyamine transporter (VPAT) that stores polyamines in secretory vesicles in astrocytes and mast cells (Hiasa et al., 2014; Takeuchi et al., 2017). As we provide evidence that spermidine permeates through the open pore of P2X receptors (Figure 2C), we propose that P2X receptors may represent an alternative polyamine pathway that is under the control of ATP. Given the importance of spermidine for the function of several other ion channels, as well as the known interactions of P2X receptors with such channels (Khakh et al., 2005; Stanchev et al., 2009; Pougnet et al., 2014; Boué-Grabot and Pankratov, 2017; Stephan et al., 2018), the question is now raised as to what extent can P2X receptors be the physiological mediator of spermidine transit.

**Table 1:** Polyamine modulation of ion channels and ligand-gated ion channels.

Ion channel/ligand-gated ion channel	Nature of the interaction with polyamines	Observations suggesting a possible implication of P2X receptors
AMPA receptors	Voltage-dependent block by intracellular polyamines (Bowie et al., 1998)	Co-localization Synaptic scaling Synaptic depression Functional interactions (Pougnet et al., 2014)
Kainate receptors	Voltage-dependent block by intracellular polyamines (Perrais et al., 2010)	Co-expression of kainate receptors and P2X receptors in some populations of neurons (Lucifora et al., 2006)
NMDA receptors	Potentiation by extracellular polyamines, existence of a polyamine binding site (Han et al., 2008; Mony et al., 2011)	Modulation of NMDA-dependent plasticity (Boué-Grabot and Pankratov, 2017)
Nicotinic receptors	Binding site for polyamine-derived toxins (Bixel et al., 2001)	Interaction between nicotinic receptors and P2X receptors: cross inhibition (Khakh et al., 2005)
Inward rectifier K^+^ (Kir) channels	Voltage-dependent blockage by intracellular polyamines Existence of a polyamine binding site (Kurata et al., 2006)	*To be determined*
ASICs	Potentiation by spermine (Babini et al., 2002)	Functional interaction between P2X receptors and ASICs (Birdsong et al., 2010; Stephan et al., 2018)
TRPV1 receptors	Polyamines can act as ligands and are able to pass through TRPV1(Ahern et al., 2006)	Cross-talk between TRPV1 and P2X receptors (Stanchev et al., 2009)

By discovering spermidine permeation through P2X receptors, our results uncover a possible overlooked P2X signaling pathway that may be extended to other natural large molecules. Earlier work provided strong evidence that P2X7 receptors are sites of release of important molecules, such as ATP and excitatory amino acids (Duan et al., 2003; Pellegatti et al., 2005; Suadicani et al., 2006; Di Virgilio et al., 2018). It now remains to determine whether these signaling molecules are physically able to cross directly through the P2X7 open pore. Identification of the mechanisms involved will undoubtedly advance the biology of P2X receptors and their role in pathological states.

## Could P2X Receptors Act as a Pathway for Therapeutic Applications?

P2X receptors can act as a pathway for the entry of large molecules into cells—to what extent can this property be exploited for therapeutic applications? Two examples of P2X receptors being used as pathways for the passage of azobenzene-based molecules have been published by the team of R. Kramer. The first example is related to the control of nociception, by manipulation of neurons involved in pain signaling via voltage-gated ion channels (Mourot et al., 2012). This was carried out by the incorporation of QAQ, an azobenzene-based photoswitch bearing two quaternary ammonium moieties, into cells through the P2X pore. Control of nociception is based on the activation/deactivation process of local voltage-gated ion channels (calcium, potassium, sodium), which is provided by the geometrical switching between *trans* and *cis* isomers of QAQ. The route responsible for the entry of QAQ, which is otherwise a membrane-impermeable molecule, can be TRPV1 or alternatively P2X7 receptors (Mourot et al., 2012). In 2016, they further developed this concept of P2X-mediated membrane passage in order to resurrect light-sensitivity of retinal ganglion cells of degenerated retina in blind mice. They employed the same methodology: a charged photoswitchable molecule based on an azobenzene moiety is applied to cells and permeates the membrane via P2X receptors. This molecule is then able to act on voltage-gated ion channels, restoring the responsiveness of retinal-ganglion cells to light (Tochitsky et al., 2016).

As stated above, the presence and implication of P2X7 in many diseases could inspire new approaches, whereby the permeability of the receptor to large cations may be utilized to therapeutic effect, in the delivery of biological effectors to P2X7-expressing cells. Equally, P2X7 has been found in over-expressed levels in several types of cancers, such as chronic B lymphocytic leukemia (De Marchi et al., 2016) and thyroid papillary cancer (Solini et al., 2008). One could envisage the exploitation of these high expression levels in conjunction with the permeability of P2X7 to large molecules, to deliver anti-cancer drugs through the P2X7 pathway.

## Conclusion

For some time now, there has been much controversy surrounding the passage of large cations through the P2X pore, and a clear explanation on the mechanism of this particular permeation pathway has remained highly elusive. However, recent advances in experimental technique allow a more just study of large cation permeation. Equally, new information showing the highly sensitive nature of P2X7 function to lipid membrane composition, which can vary between cellular types, may be considered in future studies probing the permeability of P2X7 to large cations.

New important features of P2X receptor permeability are now being brought to light. First, it is important to reconsider the structure of the open state, which exhibits an immediate extended permeability, and to consider more closely the critical role of the membrane lipid environment in order to fully understand the functionality of these proteins. Secondly, their physiological importance must also be reconsidered, with the new information that they are able to allow the passage of important physiological modulators. A final interesting point is to look further into the potential application of P2X receptors as pathway for drug delivery.

## Author Contributions

LP, KD, TC and TG wrote the manuscript. JB made the figures.

## Conflict of Interest Statement

The authors declare that the research was conducted in the absence of any commercial or financial relationships that could be construed as a potential conflict of interest.
